# Response of Soil Fauna to the Shift in a Riparian Landscape along an Urban–Rural Habitat Gradient

**DOI:** 10.3390/ijerph19148690

**Published:** 2022-07-17

**Authors:** Yumei Huang, Qian Zeng, Chunlan Luo, Danju Zhang, Wenfeng Xie, Jiujin Xiao, Yang Liu, Yushi Liu, Juan Du

**Affiliations:** 1 College of Landscape Architecture, Sichuan Agricultural University, Chengdu 611130, China; hyumei@sicau.edu.cn (Y.H.); butterfly_zeng@sina.com (Q.Z.); lcl2532235988@163.com (C.L.); xwenfeng@sicau.edu.cn (W.X.); lys20191028@163.com (Y.L.); 2 Key Laboratory of Ecological Forestry Engineering of Sichuan Province, College of Forestry, Sichuan Agricultural University, Chengdu 611130, China; zdj_8080573@sohu.com (D.Z.); j.xiao@sicau.edu.cn (J.X.); sicauliuyang@163.com (Y.L.)

**Keywords:** anthropogenic effect, revetment type, riverbank, hygrophilous soil mesofauna, urbanization

## Abstract

Urbanization is accelerating worldwide, resulting in drastic alterations of natural riverbanks, which seriously affects the ecological functions and services of riparian landscapes. Our understanding of how anthropogenic activities influence soil animal communities within riparian zones is scarce. The soil fauna represents an important biotic component of the soil ecosystem and greatly contributes to soil structure and fertility formation. We investigated the richness, abundance, diversity, and distribution of soil animal groups, including macro- and mesofauna, in different riparian landscapes along an urban–rural habitat gradient. In natural riparian zones with permeable revetments, the soil fauna was richest and most abundant, mainly because of the low levels of human disturbance and the more suitable habitat conditions. Different soil animal groups responded differently to revetment type and distance from the water flow. The hygrophilous soil mesofauna, requiring a more humid environment, was more sensitive to shifts in revetment types, the location on the riverbank, and the seasons. In summer, when precipitation in the study area was highest, the abundance of the hygrophilous soil mesofauna was significantly higher than in autumn. Distance from the water flow significantly affected the abundance of the hygrophilous soil mesofauna. Our results demonstrated that hygrophilous soil mesofauna can serve as a good indicator in riparian zones, reflecting the hydrological conditions. We also observed interactions between revetment type and distance from the water flow; the distance effect was stronger in the natural riparian zone with a permeable revetment type. Our results highlight the importance of anthropogenic effects on soil ecosystem processes and functions in riparian landscapes, and the necessity of protecting and retaining the natural riverbank and native vegetation patches in riparian landscape planning and construction.

## 1. Introduction

Riparian zones represent ecotones between aquatic and terrestrial habitats. They are characterized by heterogeneous environmental conditions and various vegetation communities, playing an important role in providing ecosystem services and functions such as flow regulation, bank stabilization, refuges for local species, water purification, and green space for recreation [1,2]. Riparian zones are generally regarded as functionally unique ecosystems [1], and numerous studies have investigated the biodiversity of plants [3], birds [4], insects [2,5], and microbes [6] in these river–land interfaces. However, these ecosystems face multiple stresses such as global climate change, land-use conversion, reshaped water regimes, increasing human interference, biological invasion, and other site-specific issues which are mainly the results of increasingly expanding urbanization [7].

Urbanization is one of the most dramatic global human alterations of ecosystems and involves major changes in environmental conditions such as increased temperatures, population aggregation, and habitat fragmentation, resulting in biodiversity loss worldwide [8,9]. Native species abundance decreases with urbanization due to the scarcity of natural areas, increases in niche competition with alien species, and disturbances arising from human activity [4,8,9]. Across an urban–rural land-use gradient, riparian zones act as corridors connecting various landscapes [4] and endure the ecologically functional ramifications of urbanization [10,11,12]. Along this gradient, various types of revetment are applied for riverbank protection, which reform the original riparian structure and change the flow regime, hence altering the ecological processes of riparian ecosystems [13,14]. Based on their water permeability, revetments can be divided into impervious and permeable ones [14]. Previous studies have shown that impervious revetments influence the energy and material exchange between rivers and riparian zones [14,15]; however, the ecological effects of various revetments on the biome, especially on soil invertebrates living in riparian zones, have received limited attention.

The soil fauna is a critical component of the soil food web [16] and can directly and indirectly influence soil biogeochemical processes [17,18]. It includes macro-, meso-, and microfauna. By definition, the macrofauna has a body size larger than 2 mm, whereas the mesofauna ranges from 100 μm to 2 mm; the microfauna is smaller than 100 μm [19]. The different soil animal groups, based on body size, play different roles in soil ecosystems [20,21]. For instance, some macrofauna taxa, such as earthworms and ants, are ecosystem engineers that can alter soil physicochemical properties through their digging and mixing activities [20]. Some mesofauna taxa, such as some mites and springtails, contribute to litter decomposition by increasing the litter surface area, improving substrate quality, and, thus, accelerating microbial activities [20]. Nematodes, a typical hygrophilous soil mesofauna group [22,23], cover various trophic levels of the soil food web, influence the rates of carbon and nitrogen fluxes of ecosystems by grazing on microbial organisms and plant roots, and prey upon other faunal groups [24]. Additionally, some microfauna taxa, such as protozoa, affect nutrient cycling by regulating the microbial population [25,26]. Given their relatively sedentary life mode and susceptibility to microenvironmental changes, the soil fauna can serve as a bioindicator to evaluate the influences of land-use alteration and management activities on soil functions [27,28]. Hodson et al. examined nematode assemblages in relatively undisturbed riparian oak woodlands and found that the complexity of the nematode community was higher than in those riparian corridors with intensive agriculture [29]. Gallardo et al. found that distance to the river was one of the most important determinants of macroinvertebrate composition due to the lateral hydrological connectivity [30]. Despite an increase in studies on soil faunal diversity in this ecological hot spot, the understanding of the responses of various soil animal groups to revetment type in a riparian landscape along an urbanization gradient is still limited.

In this context, we aimed to identify the distribution patterns of various soil animal groups along an urban–rural gradient in riparian landscapes and test the combined effects of revetment type (correlated with urbanization gradient), location on the riverbank, and season on soil animal groups in western China. We hypothesized that (1) the abundance, richness, and community structure of the soil fauna varies along urban–rural landscape gradients, and (2) soil animal groups with different moisture preferences respond differently to the revetment type and location on the riverbank. Additionally, we expected some seasonal dynamics in soil animal community composition.

## 2. Materials and Methods

### 2.1. Field Site

**Figure 1:**
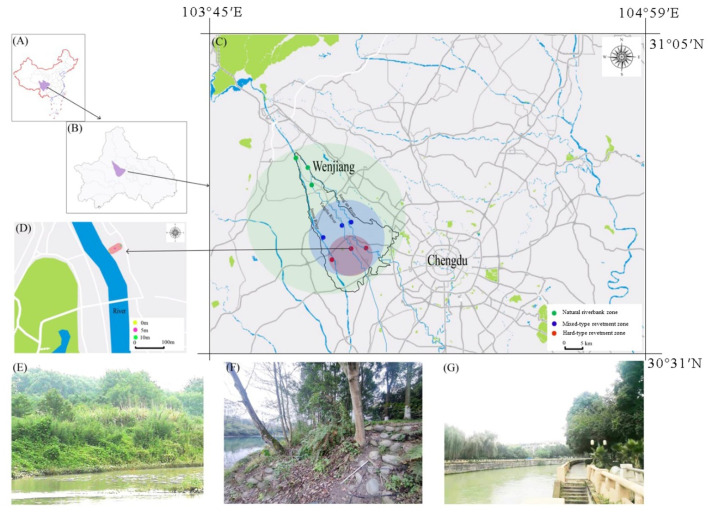
Distribution of the sampling plots along three main rivers in Wenjiang district. Sichuan Province is located in the southwest of China (**A**); Wenjiang is in the middle of Chengdu (**B**). Different urban–rural habitats are marked with round color blocks (**C**). Green color block represents a rural habitat, blue color block represents a suburban habitat, red color block represents an urban habitat (**C**). All sampling plots are marked with colored dots (**C**,**D**). Green dots represent a natural riverbank zone in a rural habitat, blue dots represent a mixed-type revetment zone in a suburban habitat, and red dots represent a hard-type revetment zone in an urban habitat (**C**). Yellow dots, purple dots, and black dots represent the sampling locations with different distances from the edge of the riverbank in each type of revetment zone (**D**). (**E**) Natural riverbank zone (NR); (**F**) mixed-type revetment zone (MR); (**G**) hard-type revetment zone (HR). Image (**C**) is exported from Planning Cloud (www.guihuayun.com, accessed on 27 October 2021).

The study was carried out in Wenjiang district (30°62′–30°87′ N, 103°69′–103°94′ E), located in the heart of Chengdu Plain, Sichuan Province, China. The region is characterized by a humid subtropical monsoonal climate, with a mean annual air temperature of 16 °C and a mean annual precipitation of 1200 mm. The rainfall in summer usually accounts for 70% of that of the entire year (https://rp5.ru/, accessed on 27 October 2021). The soil was classified as Ochri-Aquic Cambosols in the Chinese soil taxonomy, with an average pH of 6.64 and soil bulk density of 1.25 g·cm^−3^ (Table A1). Based on urban planning, distance from the city center, population density, vegetation characteristics, and a preliminary field survey, three riparian landscapes—including a natural riverbank zone (NR), a mixed-type revetment zone (MR), and a hard-type revetment zone (HR)—along an urban–rural gradient were selected to investigate the responses of the soil fauna to shifts in the revetment (or riverbank) type (“revetment type” is used in the following text for simplicity); these were located at three main rivers in Wenjiang district (Figure 1, Table A2). The “HR” plots, located in the city, with impervious masonry, fewer natural meanders, and more exotic or cultivated plants, showed the highest degree of human disturbance. The “MR” plots, located in suburban areas, were usually composed of stones and soil, resulting in some water permeability. The anthropogenic interferences of “MR” plots were usually at a medium level due to less human activity. The “NR” plots, located in rural areas, showed the lowest degree of disturbance and were characterized by natural habitats with abundant native and spontaneous vegetation. Each riparian landscape plot was replicated three times (located at three main rivers), with a total of nine sampling plots; in each plot, three sampling locations with different distances from the edge of the riverbank (0–5–10 m) were selected to assess the effects of water flow on various soil animal groups. A distance of 0 m refers to sampling in an area (50 × 50 cm) very close to the edge. In all sampling plots, iButton thermometers (Maxim Integrated, San Jose, CA, USA) were arranged to record the soil temperature.

### 2.2. Collection and Identification of the Soil Fauna

For the convenience of practical research, the classification methods for soil animals according to their body size are generally used to determine their functions in soil ecosystems [20,21], and our study was no exception. Soil macrofauna taxa, such as earthworms, ants, spiders, and beetles, acting as ecosystem engineers or predators, are generally sampled via hand-sorting. Generally, the soil mesofauna is divided into two groups according to the extracting methods [22,23]. Among them, microarthropods are generally extracted using Tullgren funnels, and the hygrophilous mesofauna is generally extracted using Baermann funnels. Considering the effects of the hydrological conditions in riparian landscapes on the soil fauna, we classified the soil fauna into three groups (macrofauna, microarthropods, and hygrophilous mesofauna), based on body size and habitat preference. 

Soil sampling was conducted in nine sampling plots for soil fauna extraction and analyses in summer (mid-July) and autumn (mid-October) of 2020, respectively. Overall, 162 soil samples were collected in each season (9 sampling plots × 6 soil samples × 3 soil animal groups). From each sampling plot, six blocks of soil (30 × 30 cm, 5 cm deep) were dug up and placed in a white porcelain tray for hand-sorting of the soil macrofauna. The macrofauna individuals picked from the soil were preserved in 75% ethanol and transported to the laboratory for further identification. Soil macrofauna abundance was expressed as individual density per square meter. For microarthropods, seven soil cores from the 0–5 cm soil layer were collected randomly (the sampling depth of soils was only 5 cm due to the thinner soil layers of urban soils) using a soil corer 2 cm in diameter, and mixed together to obtain one composite sample, with a total of six composite samples at each sampling plot. For the hygrophilous mesofauna, five soil cores from the 0–5 cm soil layer were collected randomly using a soil corer 2 cm in diameter, and mixed to obtain one composite sample, with a total of six composite samples at each sampling plot.

All soil samples were placed into nylon bags, stored in a cooler, immediately transported to the laboratory, and processed within 48 h. For the extraction of microarthropods, all Tullgren funnels were covered with 1 mm mesh to prevent springtails from escaping. The total period of specimen extraction for microarthropods was 48 h. For the hygrophilous soil mesofauna (here, mainly nematodes, also including a few enchytraeids and Diptera larvae), the extraction periods with Baermann funnels were 4, 12, 24, and 48 h. Most of the enchytraeids were collected in the 4 h collection period. The final data for each soil sample using Baermann funnels were the sum of the results of four extraction periods. During the entire extraction process, the air temperature ranged between 35 and 40 °C, regardless of the funnel type. All specimens extracted from the soil were preserved in 75% ethanol. Macrofauna individuals retrieved from the field and mesofauna individuals extracted from funnels were counted and identified to the family or subfamily level under a microscope. Based on their different ecological functions, larvae and adult specimens were classified into different groups. For identification, *Pictorial Keys to Soil Animals of China* [23] was used.

### 2.3. Vegetation Investigation

To capture the composition and diversity of the vegetation in the different riparian landscapes, nine tree quadrats (3 riparian landscapes × 3 replicates), 10 × 10 m each, were established at the soil fauna sampling plots in summer (mid-July) and autumn (mid-October) of 2020. Five shrub quadrats (2 × 2 m) and five herb quadrats (1 × 1 m) were set up at two diagonals and the center point of each tree quadrat [31]. The species name, DBH (diameter at breast height), tree height, height below branches, crown depth, and canopy density of the trees were recorded in each tree quadrat. In the shrub and herb quadrats, the species names, individual number, height, coverage, and origin of the plants were recorded. Plant identification was based on the “*Chinese Plant Illustrated Book*” [32].

### 2.4. Soil Properties

During the field sampling in summer and autumn, undisturbed soil samples from the soil surface were collected using a soil corer 5 cm in diameter at each sampling plot and transported to the laboratory for soil bulk density (SBD) analysis [33]. Six soil samples (about 1 kg each) from the 0–5 cm layer were collected from each sampling plot with a small shovel, placed in airtight polyethylene bags, and brought to the laboratory. Subsequently, they were air-dried and ground to pass through 2.0 mm sieves for the determination of soil pH, ammonium nitrogen, available phosphorus, and available potassium. The soil water content (WC) was determined gravimetrically by oven drying the freshly collected soil at 105 °C to a constant mass for 48 h [34]. The soil pH was determined using a digital pH analyzer. After calibration of the pH analyzer, 10 g of soil and 25 mL of distilled water were shaken in a test tube and precipitated for 30 min. Subsequently, the pH of the soil slurry (1:2.5, soil/distilled water, [*w*/*v*]) was determined [35]. Ammonium nitrogen (AN) was determined using the phenol-hypochlorite method [36], and available phosphorus (AP) was analyzed using a spectrophotometer, following the Olsen method [37]. Available potassium (AK) was determined using an atomic absorption spectrophotometer [38]. Soil temperature at a depth of 5 cm (iButton thermometers) and air temperature were recorded at each sampling plot both in summer and autumn.

### 2.5. Anthropogenic Disturbance Measurement

Human traffic density was used to assess the anthropogenic disturbance degrees in various riparian landscape zones [39]. In mid-July and mid-October of 2020, anthropogenic disturbance was measured in nine sampling plots. We recorded the number of pedestrians walking across each sampling plot (10 × 10 m) from 8 a.m. to 8 p.m. and calculated the mean values and standard deviations for human traffic density per hour.

### 2.6. Calculation and Statistical Analysis

Soil fauna richness and abundance were measured using the taxonomic group number and individual density per square meter, respectively; the density-group index (*DG*) was used to determine the soil fauna diversity [40]. The diversity of the vegetation in each sampling quadrat was measured using the Shannon index (*H′*), the Pielou index (*J*), and the Simpson index (*D*).

The equations were as follows:

DG=(S/G)∑i=1sNiFiNimaxF

$$P_{i}=N_{i}/N$$

$$H^{\prime }=-\sum_{i=1}^{s}P_{i}\ln P_{i}$$

$$D=\sum_{i=1}^{s}P_{i}^{2}$$

$$J=H^{\prime }/\ln S$$

For soil fauna, *S* is the taxonomic group number in each habitat; *G* is the total taxonomic group number in all habitats in each season; *N_i_* is the individual number of family *i* collected in each habitat; and *N_imax_* is the maximum individual number of family *i* in all habitats [40]; *F_i_/F* is the frequency of family *i*.

For vegetation, *P_i_* is the ratio of the individual number of species *i* to the individual number of all species in each quadrat [41]; *N_i_* is the individual number of species *i*; *N* is the individual number of all species in each quadrat; *S* is the number of species in each quadrat. According to the vertical structure of the community, the diversity indices of the tree layer, shrub layer, and herb layer should be weighted when calculating the total diversity index of the whole community [42,43], using the following equation:

*W_i_* = (*C_i_*/*C* + *H_i_*/*H*)/2,

where *C_i_* is the coverage of the specific growth form, and *i* represents the growth form of vegetation including tree layer, shrub layer, and herb layer; *C* is the sum of *C_i_*; *H_i_* is the average thickness of the leaf layer for the specific growth form; and *H* is the sum of *H_i_*. The thickness of the leaf layer for trees was calculated using 1/3 of the height of the tree layer, whereas the shrub layer was 1/2 and the herb layer was 100%. *Wi* is the weighted parameter of the diversity index of the *i*th growth form.

The community diversity index was calculated as follows:

$$\mathrm{Community}\;\mathrm{diversity}\;\mathrm{index}=\sum W_{i}\;\times \;\mathrm{Diversity}\;\mathrm{index}\;\mathrm{of}\;\mathrm{layer}\;i$$

All statistical analyses were performed using R v. 4.1.2 [44]. Generalized linear models were performed using the *MASS* package and the following multiple comparisons using the *multcomp* package [45]: non-metric multidimensional scaling (NMDS), detrended correspondence analysis (DCA), canonical correspondence analysis (CCA), and redundancy analysis (RDA) were performed using the *vegan* package [46], and indicator species analysis (ISA) was performed using the *indicspecies* package [47]. Richness and abundance data were analyzed using generalized linear models with negative binomial distribution due to the overdispersion of the count data and the Gaussian distribution for the diversity indices. A Student’s *t*-test was applied to examine the seasonal effects on the richness, abundance, and diversity indices of the soil fauna. Soil physical properties were analyzed using one-way analysis of variance (ANOVA), and the data were first tested for normal distribution and homogeneity of variance. If the data did not meet the requirements, a logarithmic, square root, or arcsine transformation was performed, followed by multiple comparisons using Tukey’s HSD method. The NMDS using the Bray–Curtis distance was performed based on the individual numbers of soil animals to visualize the differences in communities among three revetment types, three distances, and two seasons. Permutational multivariate analysis of variance (PERMANOVA) with 1000 permutations was performed to test the effects of revetment type and distance gradient on the community structure of the soil fauna in each season. To select the best analysis for the associations between soil animal community and environmental factors—including soil water content, soil bulk density, soil pH, ammonium nitrogen, available phosphorus, available potassium, soil temperature, and air temperature—DCA was performed first to calculate the gradient length [48]. The longest gradients were >4 for all analyses except for the hygrophilous mesofauna in summer. Therefore, CCA was used for all soil animal groups in autumn, as well as the macrofauna and microarthropods in summer; RDA was used for the hygrophilous mesofauna in summer (the longest gradient was <3). Only statistically significant environmental variables were plotted in CCA and RDA. The ISA based on individual numbers was conducted using 999 permutations to determine whether there were some specific families that may reflect the characteristics of environmental conditions such as revetment type, distance gradient, and season. In the ISA, both the abundance and frequency of soil animals were taken into account, and statistics were provided for each indicator taxon [49].

## 3. Results

### 3.1. Soil Fauna Abundance

**Figure 2:**
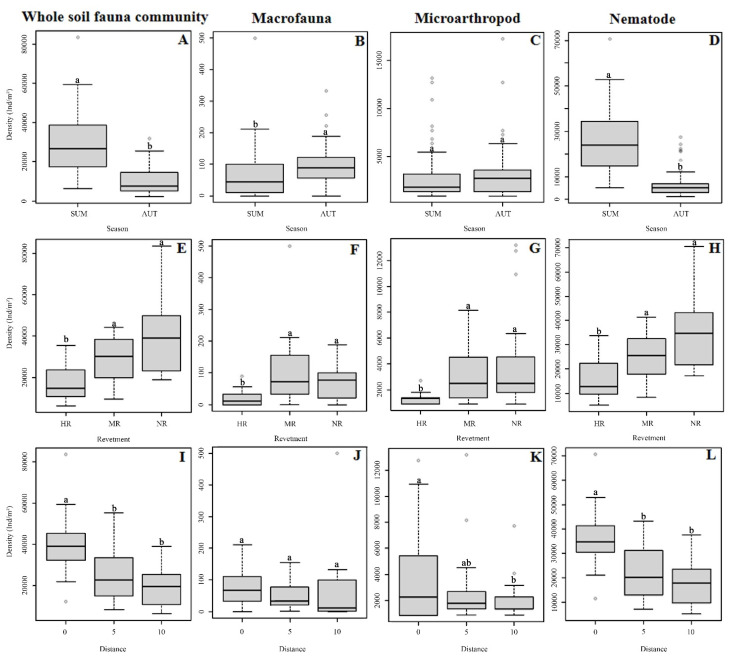
Abundance of soil animal groups in two seasons (**A**–**D**) and abundance of soil animal groups of three revetment types (**E**–**H**) and three distances (**I**–**L**) in summer. Each box plot displays the median (bold lines), the interquartile range (box), minimum and maximum values (whiskers), and outliers (circles). Different lower-case letters indicate significant differences among revetment types or distances (*p* < 0.05). The plots of three revetment types and distances in autumn are not presented due to non-significance. NR = natural riverbank zone, MR = mixed-type revetment zone, HR = hard-type revetment zone; SUM = summer, AUT = autumn; 0, 5, and 10 represent the different distances (m) from the land–water boundary. It is the same below.

Soil fauna abundance varied significantly among the different revetment types, distances, and seasons (Table 1, Figure 2). Specifically, compared with autumn, in summer, the abundance levels of the soil macrofauna, microarthropods, and hygrophilous mesofauna differed more significantly among the three revetment types and the three distances (Table 1). In most plots, soil fauna abundance was higher in the natural riverbank zone (located in rural areas with low urbanization levels) than in hard-type revetment zone (located in urban areas with high urbanization levels); moreover, soil fauna abundance in plots closer to the water flow was generally higher than that in plots further away from the water (Figure 2, Table A3). Within the same revetment type and the same distance, densities of the entire soil fauna community and the hygrophilous mesofauna tended to be higher in summer than in autumn, whereas for macrofauna, the opposite trend was observed. This verifies the existence of seasonal effects and different responses of soil animal groups to environmental shifts (Table A3). Additionally, regarding soil fauna abundance in summer, a significant interaction between revetment type and distance gradient was found (Table 1). For instance, soil fauna abundance in the natural riverbank zone (NR) differed significantly throughout the distance gradient in summer, whereas in the mixed-type revetment zone (MR) and the hard-type revetment zone (HR), these differences were less pronounced (Table A4).

### 3.2. Diversity and Community Structure

**Figure 3:**
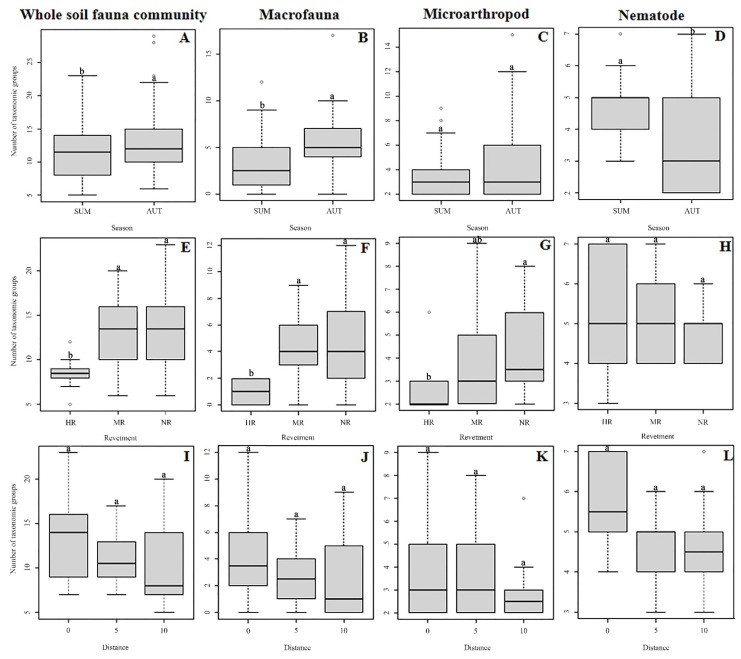
Richness of soil animal groups in two seasons (**A**–**D**) and richness of soil animal groups of three revetment types (**E**–**H**) and three distances (**I**–**L**) in summer. Taxonomic group number is the number of families, except insect larvae, which were at the order level. Each box plot displays the median (bold lines), the interquartile range (box), minimum and maximum values (whiskers), and outliers (circles). Different lower-case letters indicate significant differences among revetment types and distances (*p* < 0.05). The plots of three revetment types and distances in autumn are not presented due to non-significance.

Overall, soil animals from 191 families were collected and identified, including soil macrofauna, microarthropods, and hygrophilous mesofauna (Table A5). Generally, the richness of the entire soil fauna community was significantly higher in autumn, with a rich leaf litter layer (148 families), than in summer (119 families) (Table A5, Figure 3). Hygrophilous mesofauna richness was significantly higher in summer than in autumn (Figure 3). In NR, soil fauna richness was highest, followed by MR, whereas it was lowest in HR (Table A5). Significant differences were found between natural riverbank zones and hard-type revetment zones (Figure 3). Soil fauna richness was highest at 0 m (Table A5), although this difference was not statistically significant (Table 1, Figure 3). The interaction effect of revetment type and distance gradient on soil fauna richness was weaker than that on abundance, and was only observed for the entire soil fauna community and the macrofauna community in summer (Table 1).

The *DG* indices provided specific diversity information about the soil fauna in different revetment types, distances, and seasons (Table 2). In general, in summer, the indices were more significant compared to autumn. Additionally, the indices for the entire soil fauna community, the macrofauna, and the hygrophilous mesofauna were significantly higher in more natural riverbank zones than in highly artificial ones. Furthermore, the effect of revetment type was stronger than that of the distance gradient.

**Figure 4:**
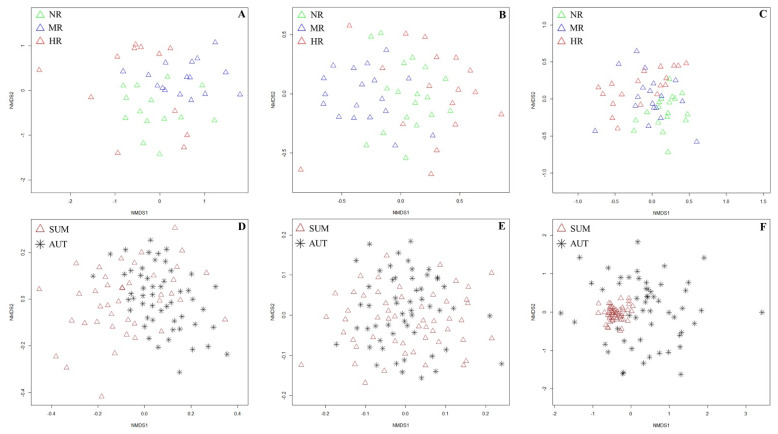
Non-metric multidimensional scaling (NMDS) based on Bray–Curtis distances visualizing the distributions of soil fauna community of different revetment types and different seasons: (**A**) distribution of three revetment types for soil macrofauna in summer; (**B**) distribution of three revetment types for microarthropods in summer; (**C**) distribution of three revetment types for the hygrophilous mesofauna in summer; (**D**) distribution of two seasons for soil macrofauna; (**E**) distribution of two seasons for microarthropods; and (**F**) distribution of two seasons for the hygrophilous mesofauna.

**Figure A1:**
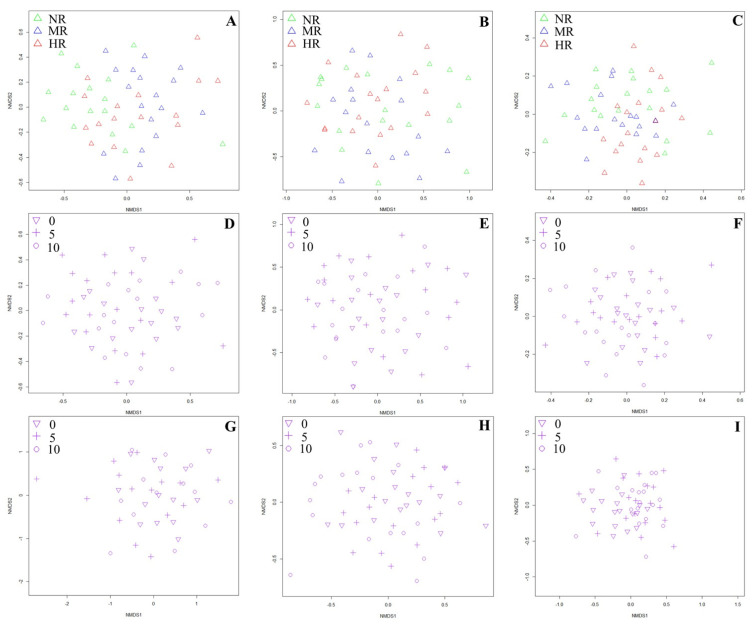
Non-metric multidimensional scaling (NMDS) based on Bray–Curtis distances visualizing the distributions of different revetment types and distances: (**A**) Distribution of three revetment types for soil macrofauna in autumn; (**B**) distribution of three revetment types for microarthropods in autumn; (**C**) distribution of three revetment types for the hygrophilous mesofauna in autumn; (**D**) distribution of three distances for soil macrofauna in autumn; (**E**) distribution of three distances for microarthropods in autumn; (**F**) distributions of three distances for the hygrophilous mesofauna in autumn; (**G**) distribution of three distances for soil macrofauna in summer; (**H**) distribution of three distances for microarthropods in summer; (**I**) distributions of three distances for the hygrophilous mesofauna in summer.

As shown via the NMDS, the soil fauna community showed more distinct separations among revetment types (Figure 4A–C) than among distances (Figure A1D–I), and more distinct separations were found in summer (Figure 4A–C) than in autumn (Figure A1A–C); this is consistent with the data shown in Table 1. Regarding the macrofauna community in summer, NMDS separations were observed among different revetment types, with only a slight overlap between NR and MR (Figure 4A). For the microarthropod community in summer, MR was separated from NR and HR, with some overlap between NR and HR (Figure 4B). Regarding the hygrophilous mesofauna community in summer, NR separated from HR, but there were some overlaps between NR and MR, as well as between MR and HR (Figure 4C). Furthermore, the NMDS separation of the hygrophilous mesofauna community was highly pronounced between summer and autumn, which was not the case for the macrofauna and microarthropods (Figure 4D–F).

Permutational multivariate analysis of variance (PERMANOVA) showed that the revetment type had significant effects on the soil fauna community structure, irrespective of the season, whereas distance only significantly affected the soil fauna community structure in summer (*p* < 0.05, Table 3).

### 3.3. Environmental Factors and Soil Fauna Communities

Canonical correspondence analysis (CCA) and redundancy analysis (RDA) were applied to evaluate the relationship between soil fauna and environmental factors (Table A1). The constrained axes explained 27.34, 17.41, and 26.08% of the variance for soil macrofauna, microarthropod, and hygrophilous mesofauna models in summer, respectively, and 16.69, 8.87, and 13.15%, respectively, in autumn (Table A6). The models for soil macrofauna, microarthropods, and hygrophilous mesofauna in summer and microarthropods in autumn were significant (*p* < 0.05, Table A6), whereas those of soil macrofauna and hygrophilous mesofauna in autumn were not significant (*p* > 0.05, Table A6).

**Figure 5:**
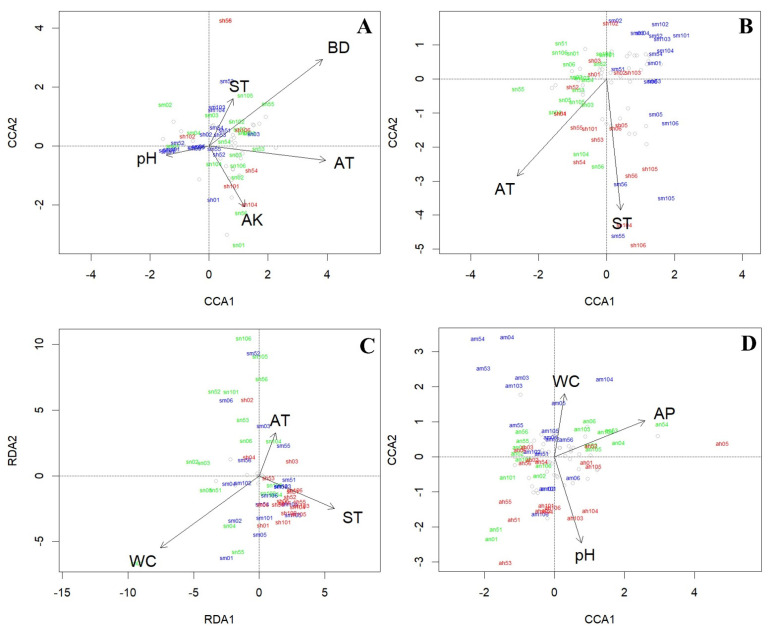
Canonical correspondence analysis (CCA) and redundancy analysis (RDA) ordination plots showing relationships between different soil fauna communities and environmental factors in two seasons. (**A**) soil macrofauna community in summer (CCA); (**B**) microarthropod community in summer (CCA); (**C**) hygrophilous mesofauna community in summer (RDA); (**D**) microarthropod community in autumn (CCA). Arrowhead indicates the direction of increase, and arrow length represents its relative influence. Different colors represent different revetment types (green = NR; blue = MR; red = HR). Grey circles represent different soil animal families. pH = soil pH; SBD = soil bulk density; AN = ammonium nitrogen N; AP = available P; AK = available K; WC = soil water content; AT = air temperature; ST = soil temperature.

Different soil animal groups responded differently to environmental factors. Temperature (air and soil) was the most important environmental factor in summer, regardless of the soil animal group (Figure 5). Specifically, for macrofauna, besides AT and ST, soil pH, BD, and AK also had significant effects on the community in summer (*p* < 0.05, Figure 5A, Table A6). For microarthropods, only AT and ST were significant factors in summer (*p* < 0.05, Figure 5B, Table A6), whereas in autumn, the significant variables were soil pH, AP, and WC *(p* < 0.05, Figure 5D, Table A6). In the case of the hygrophilous mesofauna, besides AT and ST, the community showed a stronger response to the soil water content (WC) in summer because of the high rainfall levels and their strong dependence on water (*p* < 0.05, Figure 5C, Table A6).

### 3.4. Indicator Species

For 29 families of soil fauna, there were significant associations with different revetment types, distances, and seasons (Table A7), based on indicator species analysis (ISA). For the different revetment types, a total of seventeen indicator families were found in summer, of which eight were associated with natural riverbank types, eight with the mixed-type revetment, and only one with the hard-type revetment. However, in autumn, only eight indicator families were found: three in the natural riverbank type, four in the mixed-type revetment, and one in the hard-type revetment (Table A8). In contrast, six families showed significant associations with distance and were associated with 0 m sites in summer. In autumn, a total of three indicator families were found: one in the 0 m site and two in the 5 m site.

## 4. Discussion

The temporal and spatial distribution of the soil animal community in riparian zones is a result of the interactions among revetment structure, vegetation composition, location on the riverbank, seasonal shift, and other environmental factors [50]. We hypothesized that the revetment type, which is correlated with the urbanization gradient, and the distance from the land–water boundary in different riparian landscapes induce changes in the communities of different soil animal groups by altering the hydrothermal conditions, shifting the vegetation composition, changing water availability, and varying the intensity of anthropogenic interferences.

As an important component of riparian landscapes, the type of the revetment along an urban–rural gradient significantly affected the soil fauna community in this study, which is in agreement with previous findings [2,51]. We also observed effects of distance from the water, although they were not as pronounced as those of revetment type. Different soil animal groups responded differently to changes in the riparian surroundings, which supports our second hypothesis. As expected, seasonal dynamics of the soil fauna community were also detected due to the different hydrothermal conditions.

### 4.1. Effect of Revetment Type on Soil Fauna

Our data demonstrate that the heterogeneity of microhabitats, resulting from differences in revetment types, triggered a shift in both the composition and distribution of the soil fauna, supporting our first hypothesis. Specifically, soil animals were most abundant in natural riverbank zones, and a high degree of artificiality had negative impacts on the soil fauna. The formation of riparian landscapes with different revetment types affects not only the hydrothermal conditions but also the vegetation composition on the riverbank, ultimately influencing the assemblages and activities of soil organisms through the redistribution of heat, moisture, and litter [14,52,53].

When hard-type revetments are used, such as concrete and masonry, for slope protection, the aquatic ecosystem and the riverbank ecosystem are separated; this hampers the diffusion of oxygen into the soil and the material exchange between the soil and other environmental compartments, with negative impacts on biological diversity, ecological balance, and environmental protection [13]. Flow regulation and canalization reduce the natural dynamics between the river and its banks [13] and drive changes in local habitat characteristics, thereby influencing the activities of soil dwellers [2,54].

Vegetation plays a critical role in riparian zones; for example, it maintains a suitable temperature and provides woody debris to create favorable habitats [55], and acts as a driver of soil biota composition and distribution through litter quality and quantity [56]. Apart from the complexity of vegetation, the ratio of native plant species, the size of the tree, and even the presence of certain tree species may favor soil fauna diversity at the habitat scale [2,51,57,58]. In the present study, plant diversity and the native plant ratio in the natural riverbank zone were higher than in the hard-type revetment zone (Table 1), providing more diverse foods to soil fauna; this partly contributed to the largest catch of soil fauna in the natural riverbank zone, consistent with previous findings [2,51].

Environmental changes are highly important factors that alter the structure of the soil fauna community [2,51]. Investigating how anthropogenic activities may modify the environment and affect the biome is crucial to developing reasonable revetment management [10]. In the present study, the soil fauna was strongly influenced by environmental factors such as temperature, soil moisture, soil bulk density, and pH. Water availability is a key factor that influences vegetation characteristics [59] and soil faunal distribution in riparian zones; compared with HR and MR, NR, with a higher water availability, promoted the abundance and diversity of the soil fauna, especially hygrophilous mesofauna taxa, which are more adapted to humid environments. This is in agreement with previous studies stating that natural or mixed-type revetments with better water permeability provide a relatively sustainable microhabitat and facilitate the survival of soil animals [52,53]. In addition, we found more indicator families in the natural riverbank zone than in the hard-type revetment zone, which might be, at least partly, a result of the larger species pool of the natural riverbank type. It also suggests that the hard-type revetment zone is not beneficial to the soil fauna, with fewer indicator families appearing in this habitat.

Overall, riparian landscapes with different revetments shaped the soil fauna communities through differences in water permeability, vegetation composition, and soil properties, which should be considered when retaining natural riparian habitats.

### 4.2. Effect of Distance on the Soil Fauna

Soil fauna abundance increased with decreasing distance from the land–water boundary (especially that of the microarthropods and the hygrophilous mesofauna). According to Paetzold [54], the arrival of species in a given habitat is mainly related to habitat features, such as habitat size, distance from sources, and surroundings; moreover, the influences decrease with increasing distance, which generates a distance gradient. In the gradient system, direct gradients (soil, vegetation, water, and heat gradients) represent the habitat conditions and directly influence species diversity, whereas indirect gradients (distance and landscape gradients) influence species diversity by changing the habitat conditions [50,54,60]. As both groundwater and soil moisture are partly recharged by rivers or streams, the distance from the river flow can be regarded as a proxy for water availability; hence, the influence of the river will weaken with increasing distance from the riverbank [54]. It is not surprising that the natural riverbank zone showed a more significant distance effect due to its higher water availability (Table A4). Compared with the microarthropods and the hygrophilous mesofauna, the abundance and richness of the macrofauna along the distance gradient were not obvious, whereas the hygrophilous mesofauna was more sensitive to distance; this supports our second hypothesis, which states that the different soil animal groups respond differently to environmental alterations. Macrofaunal groups, such as spiders and beetles, exhibit greater mobility and environmental tolerance than other smaller animals, which probably helps them survive under less favorable conditions [61]. Wasserstrom and Steinberger [62] found that mite diversity tended to be higher at more distant locations from the seashore and lower at closer locations in a coastal dune field in a Mediterranean ecosystem. The results showed that the gradient of environmental factors, which was generated by the distance from the land–water boundary, affected soil faunal survival, which is similar to our findings. Nematodes, a dominant group in the hygrophilous mesofauna, play an important role in soil ecological processes [24], and their abundance, community composition, and activities (i.e., locate, penetrate, hatch, and mate) can be significantly impacted by hydrothermal changes [63,64]; this is mainly because they are essentially aquatic animals that depend on the water film around soil particles for their development and movement [63,65,66]. Thus, in our study, the hygrophilous mesofauna community showed a more pronounced response to distance from the water than the other soil animal groups.

### 4.3. Effects of Seasonal Shifts on Soil Fauna

We observed significant differences in the abundance and richness of the soil fauna depending on the season (Figure 2 and Figure 3). Moreover, different revetment types and distances often had different seasonal dynamics (Figure 4 and Figure A1). Generally, the seasonal dynamics of soil animal groups are the results of the combined effects of environmental factors such as hydrothermal conditions, amount of plant litter, and soil properties [52,53,65]. In our study, both air temperature and precipitation peaked in summer, with drastic influences on soil temperature and moisture. Given the impacts of soil temperature and moisture on soil faunal feeding and development rate, climate change may have a strong impact on the abundance and community structure of the soil fauna [53,67]. According to a previous study, thermal tolerances may not only constrain where, but also when species can effectively forage for resources; this underscores the importance of climate change, as well as the heterogeneity of soil animal groups in their adaptabilities [68]. In our study, soil fauna abundance was higher in summer than in autumn, and nematodes, as the most abundant group, contributed most significantly to the distribution pattern, most likely because of the high rainfall levels in summer. However, soil fauna richness was higher in autumn than in summer, with a large contribution of macrofauna and microarthropods, which might be a result of the substantial leaf litter layer in autumn. According to Huang et al. [67], the leaf litter provides abundant food and suitable refuge for the soil fauna, and various soil animal groups, including some high-temperature sensitive species, can adapt to the mild environment; this explains the higher soil animal richness in autumn. Soil temperature and air temperature, differing in various riparian landscapes along an urban–rural gradient (Table A2) due to the urban heat island effect, significantly impacted macrofauna, microarthropod, and hygrophilous mesofauna communities in summer. In contrast, in autumn, with a more moderate climate, other soil properties such as soil pH, available potassium, and soil water content had more substantial effects (Figure 5). Furthermore, soil fauna heterogeneity along the distance gradient was more evident in summer than in autumn. This might be explained as follows: On the one hand, rainfall was higher in summer, which caused an outbreak of the hygrophilous mesofauna. Additionally, the slight differences in soil moisture along the distance gradient caused the significant differences in hygrophilous mesofauna abundance [1,63]. On the other hand, in autumn, the leaf litter layer is thicker than in summer, restricting water evaporation and increasing organic debris input, which homogenized the distance gradient plots [52].

### 4.4. Effect of Urbanization on Soil Fauna

Globally, urbanization is accelerating, resulting in the destruction of natural riverbanks and, consequently, seriously affecting the ecological functions of riparian zones [7,8]. Similar to most studies on the impacts of urbanization on biodiversity [69,70], we detected an overall negative effect on the abundance and richness of the soil animal community at high urbanization levels. Riparian landscapes are diverse along the urban–suburban–rural gradient, representing a series of distinct subsets of anthropogenic disturbance; this may reduce regional species pools or prevent some species from colonizing a site [71,72], affecting biodiversity and regional ecological processes [73]. Our results show that natural riparian zones, located in rural areas with low urbanization levels, suffered from human interference (measured using human traffic density) less than hard-type riparian zones in urban areas. In our study, the differences in revetment structure, human density, air or soil temperature, and vegetation composition were important embodiments of urbanization levels and were closely related to soil fauna diversity. The most obvious hydrological change associated with urbanization is the engineering of stream channels, in which natural features are replaced by concrete channels and streambank stabilization efforts designed to resist increased flood flows [7]. In such an approach, the riparian vegetation is replaced by an impervious or less-permeable surface, which alters the rates and pathways of water movement into, through, and out of ecosystems [7]. Walsh et al. [74] described the “urban stream syndrome” and suggested that urban riparian zones may exhibit reduced water and nutrient infiltration because natural terrestrial pathways are often bypassed due to stormwater drainage networks. Similar to soil physicochemical properties, vegetation composition is assumed to be a good predictor of the soil biota community [75]. Williams et al. [76] showed that exotic and cultivated plants were overrepresented in the species pool of urbanized regions. In our study, the proportions of exotic and cultivated plants were higher in urban areas, whereas vegetation diversity was higher in rural areas (Table A2), which is consistent with previous research. Lorenzo et al. [77] reported that numerous non-native tree species support relatively lower numbers of arthropod species than native trees, most likely because they excrete allelopathic compounds that harm native plants and some soil organisms. Overall, the occurrence of exotic plant species alters ecosystem structure and function, community assemblage, and native species interactions [78]. In this context, it is not surprising that soil fauna abundance and richness in artificial riparian landscapes with higher urbanization levels were lower than in natural riparian landscapes.

## 5. Conclusions

This work provides specific information about the changes in soil animal community traits in riparian landscapes across an urban–rural gradient. Compared with artificial and semi-natural riparian landscapes, the natural riparian landscape in rural areas had the highest soil fauna diversity because of the better permeability of the revetment structure, a less significant urban heat island effect, higher plant diversity, a higher ratio of native vegetation, and lower anthropogenic disturbance. Different soil animal groups responded differently to the revetment types, locations on the riverbank, and seasonal changes. Hygrophilous mesofauna taxa, which are adapted to humid habitats, appeared more sensitive to the environmental shift in riparian landscapes, especially the hydrological conditions. We observed interactions between revetment type and distance from the water flow, suggesting that the distance effect was stronger in natural riparian landscape with a permeable revetment type. Overall, the data presented herein demonstrate that protecting and retaining permeable revetment and native vegetation are beneficial to soil animal groups and, consequently, to riparian soil ecosystem processes and functions. With the acceleration of urbanization, natural riparian landscapes are subjected to substantial changes, frequently resulting in biodiversity loss and ecosystem degradation. Balancing ecological functions and flood control practicability, as well as protecting or restoring riparian ecosystem services under the urbanization scenario, are challenging for policymakers, urban planners, and landscape designers. Controlled experiments and long-term studies are, therefore, needed to further reveal the biological mechanisms of nutrient cycling and fertility maintenance in urban riparian zones.

## Figures and Tables

**Table 1:** Results of testing for the effects of revetment type, distance, and their interactions on soil fauna abundance and richness. *χ*^2^ statistics for the likelihood ratio test and *p* values are reported.

Season	Factor		Entire Soil Fauna Community ^a^		Macrofauna		Microarthropods		Hygrophilous Mesofauna	
			Density	Richness	Density	Richness	Density	Richness	Density	Richness
Summer	Type	*χ*2	23.663	17.977	13.535	22.258	24.786	8.285	20.004	0.290
		*p*	**0.000**	**0.000**	**0.001**	**0.000**	**0.000**	**0.016**	**0.000**	0.865
	Distance	*χ*2	20.114	5.801	0.593	1.838	5.338	1.679	20.467	2.376
		*p*	**0.000**	0.055	0.743	0.399	0.069	0.432	**0.000**	0.305
	Type × Distance	*χ* ^2^	63.697	27.845	18.911	28.294	35.197	11.178	59.865	4.472
		*p*	**0.000**	**0.001**	**0.015**	**0.000**	**0.000**	0.192	**0.000**	0.812
Autumn	Type	*χ* ^2^	0.255	0.233	1.551	0.499	1.450	0.624	1.130	0.281
		*p*	0.880	0.890	0.461	0.779	0.484	0.732	0.568	0.869
	Distance	*χ* ^2^	3.103	2.636	3.575	1.110	5.870	4.660	2.429	1.203
		*p*	0.212	0.268	0.167	0.574	0.053	0.097	0.297	0.548
	Type × Distance	*χ* ^2^	7.093	4.139	7.073	5.923	11.795	11.245	6.249	4.056
		*p*	0.527	0.844	0.529	0.656	0.161	0.188	0.619	0.852

Note: “Type” represents revetment type; here, we used “revetment type” for all sampling plots, including natural riverbank zones, for simplicity, as below. “Distance” represents the different distances from the land–water boundary. Degrees of freedom (*df*) = 51 in all cases. Significant *p* values are shown in bold (α = 0.05). ^a^ The entire soil fauna community was an assemblage consisting of all soil animal groups, including soil macrofauna, microarthropods, and hygrophilous mesofauna. It is the same below.

**Table 2:** Multiple comparisons of *DG* indices of soil fauna communities in different revetment types, distances, and seasons.

Season	Factor		Entire Soil Fauna Community	Macrofauna	Microarthropods	Hygrophilous Mesofauna
			*DG*	*DG*	*DG*	*DG*
Summer	Type	NR-MR	ns	ns	ns	ns
		NR-HR	**0.001**	**0.001**	ns	**0.001**
		MR-HR	**0.001**	**0.001**	ns	**0.008**
	Distance	0–5	**0.001**	ns	ns	**0.001**
		0–10	**0.001**	ns	ns	**0.001**
		5–10	ns	ns	ns	ns
Autumn	Type	NR-MR	ns	ns	ns	ns
		NR-HR	ns	ns	ns	ns
		MR-HR	ns	ns	ns	ns
	Distance	0–5	ns	ns	ns	ns
		0–10	ns	ns	ns	ns
		5–10	ns	ns	ns	ns

Note: “ns” means no significant difference. Significant *p* values are shown in bold. α = 0.05. “+” means the value of revetment type and distance on the left is higher than that on the right, whereas “−” has the opposite meaning.

**Table 3:** *p* values of PERMANOVA for revetment type and distance.

Season	Entire Soil Fauna Community		Macrofauna		Microarthropods		Hygrophilous Mesofauna	
	Type	Distance	Type	Distance	Type	Distance	Type	Distance
Summer	**0.001**	**0.001**	**0.001**	0.945	**0.001**	0.463	**0.001**	**0.001**
Autumn	**0.001**	0.427	**0.001**	0.403	**0.002**	0.392	**0.010**	0.709

Note: Significant *p* values are shown in bold (α = 0.05).

## Data Availability

The data presented in this study are available on request from the corresponding author. The data are not publicly available due to privacy.

## Appendix A

**Table A1:** Values of environmental variables (mean ± sd) of different revetment types and distances.

Factor	Summer						Autumn					
	Type			Distance			Type			Distance		
	NR	MR	HR	0	5	10	NR	MR	HR	0	5	10
pH (H_2_O)	6.78 ± 0.53 Aa	6.42 ± 0.78 Aa	6.59 ± 0.83 Aa	6.56 ± 0.73 Aa	6.64 ± 0.78 Aa	6.58 ± 0.71 Aa	6.82 ± 0.23 Aa	6.32 ± 0.52 Ab	6.91 ± 0.16 Aa	6.64 ± 0.46 Aa	6.68 ± 0.44 Aa	6.73 ± 0.40 Aa
SBD (g·cm^−3^)	1.29 ± 0.21 Aa	1.17 ± 0.21 Aa	1.26 ± 0.19 Aa	1.18 ± 0.18 Aa	1.27 ± 0.19 Aa	1.26 ± 0.24 Aa	1.24 ± 0.18 Aa	1.23 ± 0.21 Aa	1.31 ± 0.18 Aa	1.21 ± 0.16 Aa	1.33 ± 0.20 Aa	1.24 ± 0.19 Aa
AN (mg·kg^−1^)	18.3 ± 4.01 Ba	23.0 ± 8.28 Ba	31.2 ± 26.6 Aa	24.9 ± 24.9 Ba	23.7 ± 13.8 Ba	23.8 ± 8.3 Ba	28.8 ± 5.87 Ab	42.9 ± 29.0 Aa	28.0 ± 10.3 Ab	29.0 ± 6.1 Aa	40.5 ± 29.5 Aa	38.3 ± 11.7 Aa
AP (mg·kg^−1^)	39.4 ± 30.3 Aa	47.5 ± 24.1 Aa	44.1 ± 27.3 Aa	43.2 ± 30.4 Aa	37.1 ± 20.4 Aa	50.8 ± 29.1 Aa	37.3 ± 25.0 Aa	28.4 ± 9.96 Ba	29.0 ± 18.8 Ba	35.0 ± 20.8 Aa	30.9 ± 19.7 Aa	28.8 ± 16.9 Ba
AK (mg·kg^−1^)	150.8 ± 74.4 Aab	112.5 ± 41.3 Ab	160.6 ± 57.2 Aa	120.1 ± 62.8 Aa	147.1 ± 51.2 Aa	156.8 ± 67.4 Aa	171.0 ± 97.5 Aa	141.3 ± 71.5 Aa	131.2 ± 82.5 Aa	149.2 ± 79.9 Aa	137.7 ± 82.0 Aa	156.6 ± 95.0 Aa
WC (%)	23.4 ± 5.5 Aa	23.1 ± 4.6 Aa	20.0 ± 6.3 Aa	24.4 ± 5.5 Aa	22.1 ± 5.1 Aab	20.0 ± 5.6 Ab	20.9 ± 4.4 Aa	19.7 ± 5.1 Aa	15.3 ± 6.5 Bb	21.6 ± 4.5 Ba	18.6 ± 5.1 Bab	15.7 ± 6.3 Bb
AT (°C)	26.4 ± 2.18 Aa	22.4 ± 2.53 Ab	25.5 ± 2.59 Aa	24.7 ± 3.09 Aa	24.8 ± 2.95 Aa	24.8 ± 3.00 Aa	16.6 ± 0.59 Ba	16.7 ± 0.42 Ba	16.5 ± 0.80 Ba	16.4 ± 0.65 Ba	16.6 ± 0.62 Ba	16.7 ± 0.58 Ba
ST (°C)	25.41 ± 1.36 Aa	24.33 ± 1.47 Aa	25.96 ± 1.16 Aa	25.3 ± 1.61 Aa	25.3 ± 1.79 Aa	25.6 ± 1.93 Aa	17.95 ± 4.25 Ba	18.64 ± 4.09 Ba	19.41 ± 4.45 Ba	18.2 ± 0.26 Bab	18.0 ± 0.31 Bb	18.2 ± 0.30 Ba

Note: pH = soil pH value; SBD = soil bulk density; AN = ammonium N; AP = available P; AK = available K; WC = soil water content; AT = air temperature; ST = soil temperature. Different lower-case letters indicate significant differences among revetment types or distances in the same season, and different capital letters indicate significant differences between two seasons in the same revetment type or distance; α = 0.05.

## Appendix B

**Table A2:** Information on vegetation, human traffic, and soil temperature of different revetment sampling plots.

Factor		Revetment Type		
		NR	MR	HR
Human traffic density (ind.·h^−1^·100 m^−2^)	Summer	1.38 ± 0.39 c	4.90 ± 0.79 b	20.15 ± 1.82 a
	Autumn	1.33 ± 0.20 c	5.36 ± 1.37 b	35.43 ± 2.47 a
Vegetation diversity index	Shannon	1.17 ± 0.28 a	1.01 ± 0.31 a	1.16 ± 0.37 a
	Pielou	0.94 ± 0.08 a	0.93 ± 0.38 a	0.87 ± 0.04 a
	Simpson	0.39 ± 0.12 a	0.53 ± 0.14 a	0.38 ± 0.11 a
Vegation composition	Exotics plants/native plants	27%	30%	39%
	Cultivated plants/spontaneous plants	3%	33%	195%
Soil temperature (°C)	June	24.08 ± 0.37 a	22.29 ± 0.23 b	24.59 ± 0.41 a
	July	27.21 ± 0.35 a	25.34 ± 0.28 b	27.26 ± 0.29 a
	August	24.93 ± 0.26 b	25.36 ± 0.33 ab	26.03 ± 0.42 a
	September	23.17 ± 0.29 b	23.64 ± 0.39 ab	24.95 ± 0.76 a
	October	17.90 ± 0.15 b	18.64 ± 0.15 a	19.15 ± 0.29 a
	November	12.77 ± 0.25 b	13.64 ± 0.05 a	14.12 ± 0.15 a

Note: Human traffic density was measured in both summer and autumn by counting the number of pedestrians who walked across each sampling plot (10 × 10 m) from 8 a.m. to 8 p.m. and calculating the mean values and standard deviation values per hour. Different lower-case letters indicate significant differences among revetment types (*p* < 0.05).

## Appendix C

**Table A3:** Density of the soil fauna (mean ± sd, ind. m^−2^) of different revetment types and distances in two seasons.

Factor		Entire Soil Fauna Community		Macrofauna		Microarthropods		Hygrophilous Mesofauna	
		Summer	Autumn	Summer	Autumn	Summer	Autumn	Summer	Autumn
Type	NR	39,334.6 ± 16,933.9 Aa	10,010.3 ± 7808.2 Ba	70.3 ± 54.7 Aa	80.2 ± 40.2 Aa	4267.7 ± 3941.5 Aa	3737.4 ± 4013.1 Aa	34,996.4 ± 14,169.5 Aa	6192.6 ± 6424.6 Ba
	MR	28,631.0 ± 10,037.7 Aa	11,048.5 ± 7390.5 Ba	108.1 ± 115.6 Aa	106.8 ± 72.0 Aa	3257.7 ± 2360.5 Aa	2802.9 ± 1666.4 Aa	25,265.4 ± 9915.8 Aa	8138.8 ± 7371.7 Ba
	HR	17,918.5 ± 9400.3 Ab	10,135.4 ± 6927.9 Ba	19.7 ± 23.1 Bb	98.7 ± 77.4 Aa	1338.4 ± 479.8 Bb	3207.1 ± 2834.2 Aa	16,560.4 ± 9381.4 Ab	6829.5 ± 5766.9 Ba
Distance	0	40,549.2 ± 15,149.3 Aa	12,057.0 ± 7534.1 Ba	76.6 ± 64.3 Aa	119.6 ± 74.4 Aa	3813.1 ± 3514.5 Aa	3232.2 ± 2193.1 Ab	36,659.6 ± 12,669.9 Aa	8704.8 ± 6804.2 Ba
	5	25,226.2 ± 12,498.7 Ab	8345.0 ± 4218.1 Ba	53.7 ± 49.6 Ba	87.1 ± 56.3 Aa	2904.2 ± 3087.3 Aab	2348.5 ± 1472.9 Aab	22,292.9 ± 10,784.9 Ab	5909.5 ± 4062.5 Ba
	10	20,084.1 ± 9677.2 Ab	10,792.1 ± 9064.6 Ba	67.8 ± 119.0 Aa	79.0 ± 59.1 Aa	2146.6 ± 1633.6 Ab	4166.7 ± 4318.5 Aa	17,869.8 ± 9275.4 Ab	6546.5 ± 7975.8 Ba

Note: Different lower-case letters indicate significant differences among revetment types or distances in the same season, and different capital letters indicate significant differences between the two seasons in the same revetment type or distance; α = 0.05.

## Appendix D

**Table A4:** Results of testing for the effects of distance gradient on soil fauna density and diversity in the same revetment type. *χ^2^* statistics for the likelihood ratio test and *p* values are reported.

Factor			Entire Soil Fauna Community		Macrofauna		Microarthropods		Hygrophilous Mesofauna	
			Density	Richness	Density	Richness	Density	Richness	Density	Richness
Summer	NR	*χ^2^*	11.688	4.967	120.850	2.292	6.646	1.767	11.285	0.092
		*p*	**0.003**	0.083	**0.000**	0.318	**0.036**	0.413	**0.004**	0.955
	MR	*χ^2^*	11.628	0.811	0.656	0.928	0.440	0.685	11.138	0.153
		*p*	**0.003**	0.667	0.721	0.629	0.803	0.710	**0.004**	0.926
	HR	*χ^2^*	15.710	2.898	4.496	2.000	0.911	0.392	15.900	3.936
		*p*	**0.000**	0.235	0.106	0.368	0.634	0.822	**0.000**	0.140
Autumn	NR	*χ^2^*	1.937	1.253	1.429	1.427	4.702	5.065	0.464	2.101
		*p*	0.380	0.534	0.489	0.490	0.095	0.079	0.793	0.350
	MR	*χ^2^*	0.958	3.905	5.925	2.484	0.343	2.448	0.784	0.208
		*p*	0.619	0.142	0.052	0.290	0.843	0.294	0.676	0.901
	HR	*χ^2^*	3.949	0.104	0.009	1.618	3.944	2.304	4.237	1.466
		*p*	0.139	0.949	0.996	0.445	0.139	0.316	0.120	0.481

Note: Degrees of freedom (*df*) = 15 in all cases. Significant *p* values are shown in bold (α = 0.05).

## Appendix E

**Table A5:** Compositions of soil fauna communities for three revetment types and three distances.

Phylum	Class	Order	Family	Summer						Autumn					
				Type			Distance			Type			Distance		
				NR	MR	HR	0	5	10	NR	MR	HR	0	5	10
Rotifera	Digononta	Bdelloidea	Philodinidae	−	−	−	−	−	−	−	−	+	−	+	+
Nemata	Secernentea	Tylenchida	Criconematidae	−	+	−	+	−	+	+	+	−	+	+	+
Nemata	Secernentea	Tylenchida	Tylenchidae	−	+	+	+	+	+	+	+	+	+	+	+
Nemata	Secernentea	Tylenchida	Pratylenchidae	+	+	+	+	+	+	+	+	+	+	+	+
Nemata	Secernentea	Tylenchida	Heteroderidae	+	−	−	+	+	+	−	−	−	−	−	−
Nemata	Secernentea	Tylenchida	Paratylenchidae	+	+	+	+	+	+	+	+	+	+	+	+
Nemata	Secernentea	Tylenchida	Nothotylenchidae	−	−	−	−	−	−	+	−	+	+	+	+
Nemata	Secernentea	Tylenchida	Aphelenchoidea	−	−	+	+	+	+	+	+	+	+	+	+
Nemata	Secernentea	Rhabditida	Rhabditidea	+	+	+	+	+	+	+	+	+	+	+	+
Nemata	Secernentea	Rhabditida	Panagrolaimidae	−	−	−	−	−	−	−	−	+	−	−	+
Nemata	Adenophorea	Dorylaimida	Carcharolaimidae	−	−	−	−	−	−	+	−	−	+	+	−
Nemata	Adenophorea	Dorylaimida	Dorylaimidae	+	+	+	+	+	+	+	+	+	+	+	+
Nemata	Adenophorea	Araeolaimida	Plectidae	+	+	+	+	+	+	+	+	+	+	+	+
Annelida	Oligochaeta	Microdrile oligochaetes	Lumbricidae	+	+	+	+	+	+	+	+	+	+	+	+
Annelida	Oligochaeta	Megadrile oligochaetes	Enchytraeidae	−	−	−	−	−	−	−	+	+	−	+	+
Annelida	Hirudinea	Arhynchobdellida	Haemadipsidae	−	−	−	−	−	−	−	+	−	+	+	−
Mollusca	Gastropoda	Mesogastropoda	Cyclophoridae	−	−	−	−	−	−	−	+	+	+	−	+
Mollusca	Gastropoda	Stylommatophora	Subulinidae	+	+	+	+	+	+	+	+	+	+	+	+
Mollusca	Gastropoda	Stylommatophora	Pupillidae	+	−	−	+	+	−	+	+	+	+	+	+
Mollusca	Gastropoda	Stylommatophora	Bradybaenieae	−	+	+	+	−	−	+	+	+	+	+	+
Mollusca	Gastropoda	Stylommatophora	Ariophantidae	−	+	+	+	+	−	−	+	−	+	−	+
Mollusca	Gastropoda	Stylommatophora	Succineidae	−	+	−	+	−	−	−	−	+	−	+	−
Mollusca	Gastropoda	Stylommatophora	Clausiliidae	−	−	−	−	−	−	−	+	−	+	−	−
Mollusca	Gastropoda	Stylommatophora	Valloniidae	−	−	−	−	−	−	−	−	+	−	+	−
Mollusca	Gastropoda	Stylommatophora	Camaenidae	−	−	−	−	−	−	−	−	+	−	+	−
Arthropoda	Arachnida	Araneae	Theridiidae	+	+	−	+	+	+	+	+	+	+	+	+
Arthropoda	Arachnida	Araneae	Thermozodia	+	−	−	+	−	−	−	−	−	−	−	−
Arthropoda	Arachnida	Araneae	Ctenizidae	+	−	−	+	+	−	−	−	−	−	−	−
Arthropoda	Arachnida	Araneae	Anyphaenidae	+	−	−	−	+	−	−	−	−	−	−	−
Arthropoda	Arachnida	Araneae	Tetragnathidae	+	−	−	+	+	−	−	−	−	−	−	−
Arthropoda	Arachnida	Araneae	Oonopidae	+	−	−	−	+	−	−	−	−	−	−	−
Arthropoda	Arachnida	Araneae	Clubionidae	+	−	−	+	−	−	−	−	−	−	−	−
Arthropoda	Arachnida	Araneae	Lycosidae	+	−	−	+	−	+	+	+	+	+	+	+
Arthropoda	Arachnida	Araneae	Oecobiidae	+	−	−	+	+	−	−	−	−	−	−	−
Arthropoda	Arachnida	Araneae	Selenopidae	+	−	−	−	−	+	−	−	−	−	−	−
Arthropoda	Arachnida	Araneae	Araneidae	+	−	−	+	−	−	−	+	−	+	−	+
Arthropoda	Arachnida	Araneae	Oxyopidae	−	−	−	−	−	−	+	−	+	−	+	−
Arthropoda	Arachnida	Araneae	Scytodidae	−	−	−	−	−	−	+	−	−	−	+	−
Arthropoda	Arachnida	Araneae	Mimetidae	−	−	−	−	−	−	+	−	−	+	−	+
Arthropoda	Arachnida	Araneae	Pholcidae	−	−	−	−	−	−	+	−	+	−	+	−
Arthropoda	Arachnida	Araneae	Liphistiidae	−	−	−	−	−	−	+	−	+	+	+	−
Arthropoda	Arachnida	Araneae	Zoridae	−	−	−	−	−	−	+	+	+	+	+	+
Arthropoda	Arachnida	Araneae	Heteropodidae	−	−	−	−	−	−	+	−	−	−	+	−
Arthropoda	Arachnida	Araneae	Filistaidae	−	−	−	−	−	−	−	+	−	+	−	−
Arthropoda	Arachnida	Araneae	Linyphiidae	−	−	−	−	−	−	−	+	−	−	−	+
Arthropoda	Arachnida	Araneae	Pisauride	−	−	−	−	−	−	−	+	−	−	+	+
Arthropoda	Arachnida	Araneae	Agelenidae	−	−	−	−	−	−	−	−	+	+	+	−
Arthropoda	Arachnida	Araneae	Atypidae	−	−	−	−	−	−	−	−	+	−	+	−
Arthropoda	Arachnida	Araneae	Ctenidae	+	+	−	+	+	+	+	−	−	+	+	+
Arthropoda	Arachnida	Araneae	Gnaphosidae	+	−	−	+	−	−	−	−	−	−	−	−
Arthropoda	Arachnida	Araneae	Philodromidae	−	+	−	−	−	+	−	−	−	−	−	−
Arthropoda	Arachnida	Parasiformes	Ameroseiidae	+	+	−	+	+	+	+	+	+	+	+	+
Arthropoda	Arachnida	Parasiformes	Epicridae	+	+	+	+	+	+	−	+	−	−	+	−
Arthropoda	Arachnida	Parasiformes	Ascidae	−	+	+	−	+	+	−	−	+	+	−	+
Arthropoda	Arachnida	Parasiformes	Veigaiidae	+	−	−	+	−	−	+	+	−	+	+	+
Arthropoda	Arachnida	Parasiformes	Macrochelidae	+	+	−	+	−	−	−	−	−	−	−	−
Arthropoda	Arachnida	Parasiformes	Parholaspididae	+	+	+	+	+	+	+	+	+	−	+	+
Arthropoda	Arachnida	Parasiformes	Laeclapidae	+	+	+	+	+	+	+	+	+	+	+	+
Arthropoda	Arachnida	Parasiformes	Ologamasidae	−	+	−	−	−	+	−	−	−	−	−	−
Arthropoda	Arachnida	Parasiformes	Pachylaelapidae	−	−	+	+	+	+	−	−	−	−	−	−
Arthropoda	Arachnida	Acariformes	Parasitidae	+	+	+	+	+	+	+	−	+	+	+	+
Arthropoda	Arachnida	Acariformes	Sejidae	+	+	−	+	−	+	−	−	−	−	−	−
Arthropoda	Arachnida	Acariformes	Microdispidae	−	+	−	+	+	−	+	+	−	+	+	+
Arthropoda	Arachnida	Acariformes	Cheylrtidae	−	−	−	−	−	−	−	−	+	+	−	−
Arthropoda	Arachnida	Acariformes	Scutacaridae	+	−	−	+	−	−	−	−	−	−	−	−
Arthropoda	Arachnida	Acariformes	Cryptognathidae	−	−	−	−	−	−	+	−	−	−	−	+
Arthropoda	Arachnida	Acariformes	Pygmephoridae	−	+	−	−	−	+	+	+	+	+	+	+
Arthropoda	Arachnida	Acariformes	Tarsonmidae	−	−	−	−	−	−	+	+	+	+	−	+
Arthropoda	Arachnida	Acariformes	Cunaxidae	+	−	−	−	+	+	−	−	−	−	−	−
Arthropoda	Arachnida	Acariformes	Erythraeidae	−	−	−	−	−	−	+	−	−	+	−	−
Arthropoda	Arachnida	Acariformes	Zetorchestidae	+	+	+	+	+	+	+	+	−	+	+	+
Arthropoda	Arachnida	Acariformes	Oribatellidae	+	−	+	+	+	−	−	+	+	+	+	+
Arthropoda	Arachnida	Acariformes	Lohmannidae	−	−	−	−	−	−	+	−	+	−	−	+
Arthropoda	Arachnida	Acariformes	Trhypochthoniidae	−	−	−	−	−	−	−	+	−	+	−	−
Arthropoda	Arachnida	Acariformes	Mochlozetidae	−	−	−	−	−	−	−	+	−	−	+	−
Arthropoda	Arachnida	Acariformes	Hypochthoniidae	−	−	−	−	−	−	−	−	+	+	−	−
Arthropoda	Arachnida	Acariformes	Liacaridae	+	+	−	+	+	+	+	−	+	+	−	+
Arthropoda	Arachnida	Acariformes	Nothridae	−	+	−	+	−	+	+	−	+	−	+	+
Arthropoda	Arachnida	Acariformes	Malaconothridae	+	−	−	−	+	−	−	−	+	+	+	+
Arthropoda	Arachnida	Acariformes	Ceratozetidae	+	+	−	+	−	+	+	−	−	−	−	+
Arthropoda	Arachnida	Acariformes	Suctobelbidae	+	+	−	+	+	+	−	−	−	−	−	−
Arthropoda	Arachnida	Acariformes	Rhagidiidae	−	−	−	−	−	−	+	−	+	+	+	−
Arthropoda	Arachnida	Acariformes	Galumnidae	−	+	−	−	+	−	+	+	+	+	+	+
Arthropoda	Arachnida	Acariformes	Carabodidae	+	−	+	−	+	+	−	−	−	−	−	−
Arthropoda	Arachnida	Acariformes	Oppiidae	+	+	+	+	+	+	+	+	+	+	+	+
Arthropoda	Arachnida	Acariformes	Scheloribatidae	−	+	−	−	+	−	−	+	+	+	−	+
Arthropoda	Arachnida	Acariformes	Oribatulidae	+	+	+	+	+	+	+	−	+	+	+	−
Arthropoda	Arachnida	Acariformes	Labidostommidae	−	−	−	−	−	−	−	−	+	−	−	+
Arthropoda	Arachnida	Acariformes	Hermanniidae	−	+	−	−	+	−	−	−	+	+	−	−
Arthropoda	Arachnida	Acariformes	Cepheidae	−	−	+	−	+	−	+	−	+	+	+	−
Arthropoda	Arachnida	Acariformes	Peloppiidae	+	−	+	+	+	−	−	+	+	+	+	−
Arthropoda	Arachnida	Acariformes	Phthiracaridae	−	−	−	−	−	−	−	−	+	+	−	+
Arthropoda	Arachnida	Acariformes	Scutoverticidae	−	−	−	−	−	−	−	−	+	−	−	+
Arthropoda	Malacostraca	Isopoda	Oniscidae	−	+	−	−	+	+	−	−	−	−	−	−
Arthropoda	Malacostraca	Isopoda	Philosciidae	−	−	−	−	−	−	+	−	−	+	+	+
Arthropoda	Malacostraca	Isopoda	Porcellionidae	−	+	+	−	+	+	+	−	+	+	+	+
Arthropoda	Diplopoda	Polyxenida	Sphaeropoeidae	−	−	−	−	−	−	+	−	−	−	+	+
Arthropoda	Diplopoda	Spirobolida	Spirobolidae	−	+	−	−	+	+	+	+	−	+	+	−
Arthropoda	Diplopoda	Julida	Julidae	−	+	−	+	+	+	+	−	+	+	+	+
Arthropoda	Chilopoda	Scutigeromorpha	Scutiger coleoptrata	−	−	−	−	−	−	+	−	−	−	+	−
Arthropoda	Chilopoda	Lithomorpha	Lithobiidae	−	+	−	+	−	+	−	−	+	+	+	+
Arthropoda	Chilopoda	Scolopendromorpha	Scolopendridae	−	−	−	−	−	−	+	+	+	+	+	+
Arthropoda	Chilopoda	Geophilomopha	Geophilidae	+	+	+	+	+	+	+	+	+	+	+	+
Arthropoda	Symphyla	Symphyla	Scoutigerellidae	−	−	−	−	−	−	+	−	+	−	−	+
Arthropoda	Symphyla	Symphyla	Scolopendrellidae	−	−	−	−	−	−	+	+	+	+	+	+
Arthropoda	Protura	Acerentomata	Protentomidae	−	−	−	−	−	−	+	+	+	+	+	+
Arthropoda	Protura	Acerentomata	Hesperentomidae	−	−	−	−	−	−	−	−	+	−	+	−
Arthropoda	Protura	Sinentomata	Sinentomidae	+	−	−	+	+	−	−	−	+	−	−	+
Arthropoda	Protura	Sinentomata	Fujientomidae	−	−	−	−	−	−	+	−	−	+	−	−
Arthropoda	Collembola	Collembola	Neanuridae	−	−	−	−	−	−	+	+	+	+	+	+
Arthropoda	Collembola	Collembola	Onychiuridae	+	−	−	+	+	+	+	+	+	+	+	+
Arthropoda	Collembola	Collembola	Sminthuridae	+	+	+	−	+	+	+	+	+	+	+	+
Arthropoda	Collembola	Collembola	Hypogastruridae	+	+	−	+	+	+	+	+	+	+	+	+
Arthropoda	Collembola	Collembola	Poduridae	+	+	+	+	+	+	+	+	+	+	−	+
Arthropoda	Collembola	Collembola	Neelidae	+	+	+	+	+	+	+	+	−	+	+	+
Arthropoda	Collembola	Collembola	Isotomidae	+	−	+	+	+	+	+	+	+	+	+	+
Arthropoda	Collembola	Collembola	Orchesellidae	−	+	−	−	−	+	+	+	+	+	−	+
Arthropoda	Collembola	Collembola	Tomoceridae	−	−	−	−	−	−	+	−	+	+	+	+
Arthropoda	Collembola	Collembola	Cyphoderidae	−	+	+	+	−	−	−	−	+	+	−	−
Arthropoda	Diplura	Diplura	Japygidae	+	−	−	−	+	−	+	+	+	−	+	−
Arthropoda	Diplura	Diplura	Campodeidae	−	+	−	+	+	+	−	−	+	+	−	−
Arthropoda	Insecta	Isoptera	Rhinotermitidae	+	−	−	+	−	−	−	−	−	−	−	−
Arthropoda	Insecta	Isoptera	Hodotermitidae	−	−	−	−	−	−	+	−	−	−	+	+
Arthropoda	Insecta	Isoptera	Kalotermitidae	−	−	−	−	−	−	−	+	+	+	+	+
Arthropoda	Insecta	Orthoptera	Tetrigoidea	−	−	−	−	−	−	−	+	−	+	+	+
Arthropoda	Insecta	Orthoptera	Grylloidea	−	−	−	−	−	−	+	−	+	−	+	+
Arthropoda	Insecta	Deramptera	Forficulidae	−	+	−	−	−	+	+	−	−	+	−	−
Arthropoda	Insecta	Deramptera	Spongiphoridae	−	−	−	−	−	−	−	−	+	+	−	−
Arthropoda	Insecta	Hemiptera	Tingidae	+	−	−	−	+	−	−	−	−	−	−	−
Arthropoda	Insecta	Hemiptera	Lygaeidae	+	−	+	+	+	+	−	+	+	+	−	+
Arthropoda	Insecta	Hemiptera	Cydnidae	+	+	−	+	+	+	−	+	+	+	+	+
Arthropoda	Insecta	Hemiptera	Hebridae	+	−	−	−	−	+	+	+	+	+	+	+
Arthropoda	Insecta	Hemiptera	Enicocephalidae	−	−	−	−	−	−	−	+	−	−	+	+
Arthropoda	Insecta	Hemiptera	Pyrrhocoridae	−	−	−	−	−	−	−	+	+	+	−	+
Arthropoda	Insecta	Hemiptera	Schizopteridae	−	−	−	−	−	−	−	−	+	−	−	+
Arthropoda	Insecta	Hemiptera	Anthocoridae	+	+	−	+	+	+	+	+	−	+	+	−
Arthropoda	Insecta	Psocoptera	Amphientomidae	+	−	+	−	+	−	−	−	−	−	−	−
Arthropoda	Insecta	Psocoptera	Archipsocidae	−	+	−	+	−	−	−	−	−	−	−	−
Arthropoda	Insecta	Psocoptera	Psocidae	−	−	−	−	−	−	−	−	+	−	+	−
Arthropoda	Insecta	Psocoptera	Sphaeropsocidae	+	−	−	−	+	−	−	−	−	−	−	−
Arthropoda	Insecta	Psocoptera	Liposcelididae	+	+	+	+	+	+	+	+	−	+	−	+
Arthropoda	Insecta	Psocoptera	Caeciliidae	−	+	−	+	−	−	−	−	−	−	−	−
Arthropoda	Insecta	Thysanoptera	Phlaeothripidae	−	−	−	−	−	−	+	+	+	+	+	+
Arthropoda	Insecta	Coleoptera	Discodomidae	+	−	−	+	−	−	−	−	−	−	−	−
Arthropoda	Insecta	Coleoptera	Hydrophilidae	+	−	+	+	+	−	−	−	−	−	−	−
Arthropoda	Insecta	Coleoptera	Carabidae	+	+	+	−	+	+	−	−	+	+	+	+
Arthropoda	Insecta	Coleoptera	Staphylinidae	+	−	+	+	+	+	+	−	−	+	−	+
Arthropoda	Insecta	Coleoptera	Georissidae	+	−	−	−	−	+	−	−	−	−	−	−
Arthropoda	Insecta	Coleoptera	Lagriidae	+	−	−	−	−	+	−	−	−	−	−	−
Arthropoda	Insecta	Coleoptera	Silphidae	+	−	−	−	−	+	−	−	−	−	−	−
Arthropoda	Insecta	Coleoptera	Elateridae	+	−	−	−	−	+	−	−	+	+	+	−
Arthropoda	Insecta	Coleoptera	Endomychidae	−	−	−	−	−	−	+	−	−	+	−	−
Arthropoda	Insecta	Coleoptera	Languriidae	−	−	−	−	−	−	+	−	−	+	−	−
Arthropoda	Insecta	Coleoptera	Cucujidae	−	−	−	−	−	−	+	+	−	+	+	+
Arthropoda	Insecta	Coleoptera	Mycetoptoophagidae	−	−	−	−	−	−	+	+	+	+	+	+
Arthropoda	Insecta	Coleoptera	Scarabaeidae	−	+	−	−	−	+	−	−	+	+	−	−
Arthropoda	Insecta	Coleoptera	Lucanidae	−	+	−	−	−	+	−	−	+	+	−	−
Arthropoda	Insecta	Coleoptera	Cicindelidae	−	+	−	+	−	+	−	−	−	−	−	−
Arthropoda	Insecta	Coleoptera	Biphyllidae	−	−	−	−	−	−	−	+	−	+	−	+
Arthropoda	Insecta	Coleoptera	Nitidulidae	−	+	−	−	−	+	−	−	−	−	−	−
Arthropoda	Insecta	Coleoptera	Cleridae	+	−	−	−	+	+	−	−	−	−	−	−
Arthropoda	Insecta	Coleoptera	Archeocrypticidae	+	−	−	+	−	−	−	−	−	−	−	−
Arthropoda	Insecta	Coleoptera	Chrysomelidae	+	−	−	+	−	−	−	−	−	−	−	−
Arthropoda	Insecta	Coleoptera	Scydmaenidae	+	−	−	−	−	+	−	−	+	−	+	+
Arthropoda	Insecta	Coleoptera	Pselaphidae	−	−	−	−	−	−	+	−	−	−	+	+
Arthropoda	Insecta	Coleoptera	Curculionidae	−	−	−	−	−	−	−	+	−	+	−	−
Arthropoda	Insecta	Coleoptera	Throscidae	−	−	−	−	−	−	−	−	+	−	−	+
Arthropoda	Insecta	Coleoptera	Silvanidae	+	−	−	−	−	+	+	+	−	+	+	+
Arthropoda	Insecta	Coleoptera	Carabidae larvae	−	+	−	−	−	+	−	+	−	+	−	−
Arthropoda	Insecta	Coleoptera	Lampyridae	−	−	+	−	+	−	−	−	−	−	−	−
Arthropoda	Insecta	Lepidoptera	Geometridae larvae	+	−	−	+	−	−	+	−	−	−	−	+
Arthropoda	Insecta	Lepidoptera	Hepialide larvae	+	−	−	+	−	−	−	−	−	−	−	−
Arthropoda	Insecta	Lepidoptera	Pieridae larvae	−	−	−	−	−	−	−	+	−	−	+	−
Arthropoda	Insecta	Lepidoptera	Noctuidae larvae	−	−	−	−	−	−	−	+	−	−	+	−
Arthropoda	Insecta	Lepidoptera	Notodontidae larvae	−	−	+	+	−	−	−	−	−	−	−	−
Arthropoda	Insecta	Diptera	Ceratopogonidae larvae	+	−	−	+	−	−	−	+	−	+	+	−
Arthropoda	Insecta	Diptera	Dolichopodidae larvae	+	−	−	+	−	−	−	−	−	−	−	−
Arthropoda	Insecta	Diptera	Asteiidae larvae	+	−	−	+	−	−	−	−	−	−	−	−
Arthropoda	Insecta	Diptera	Mycetophilidae larvae	−	−	−	−	−	−	+	−	−	+	−	+
Arthropoda	Insecta	Diptera	Scatopsidae larvae	−	+	−	−	+	+	+	−	−	−	+	+
Arthropoda	Insecta	Diptera	Chironomidae larvae	−	−	−	−	−	−	+	+	−	+	+	+
Arthropoda	Insecta	Diptera	Tipulidae larvae	−	−	−	−	−	−	−	+	−	+	−	−
Arthropoda	Insecta	Diptera	Empididae larvae	−	−	−	−	−	−	+	+	+	+	+	−
Arthropoda	Insecta	Diptera	Phoridae	−	−	−	−	−	−	−	−	+	−	+	−
Arthropoda	Insecta	Diptera	Anisopodidae larvae	+	−	−	+	−	−	−	−	+	−	−	+
Arthropoda	Insecta	Diptera	Sciaridae larvae	−	−	−	−	−	−	+	−	−	+	−	−
Arthropoda	Insecta	Diptera	Hesperinidae larvae	−	+	−	−	+	−	−	−	−	−	−	−
Arthropoda	Insecta	Diptera	Limoniidae larvae	−	−	−	−	−	−	−	+	+	+	+	+
Arthropoda	Insecta	Hymenoptera	Ponerinae	+	+	+	+	+	+	−	−	−	−	−	−
Arthropoda	Insecta	Hymenoptera	Leptanillinae	−	+	−	+	+	−	+	+	+	+	+	+
Arthropoda	Insecta	Hymenoptera	Dorylinae	−	+	−	+	−	+	−	+	+	+	+	−
Total taxonomic group number				80	66	40	79	71	72	87	79	96	106	98	97

Note: "+" indicates that there are soil animals of this family in the sampling plot; "-" indicates that there are no soil animals of this family in the sampling plot.

## Appendix G

**Table A6:** Results of the canonical correspondence analysis (CCA) and redundancy analysis (RDA) of soil fauna. Significant values are marked in bold.

Season	Soil Animal Group	Variability Explained (%)	*p* Values										
			Reduced Model	Axes		pH	SBD	AN	AP	AK	WC	AT	ST
				CCA1 (RDA1)	CCA2 (RDA2)								
Summer	Macrofauna	27.34%	**0.001**	**0.001**	0.113	**0.001**	**0.002**			**0.032**		**0.001**	**0.005**
	Microarthropods	17.41%	**0.033**	**0.003**	0.607							**0.001**	**0.003**
	Hygrophilous mesofauna	26.08%	**0.002**	**0.001**	0.436						**0.002**	**0.038**	**0.004**
Autumn	Macrofauna	16.69%	0.089	0.379	0.642								
	Microarthropods	8.87%	**0.002**	**0.027**	0.071	**0.039**			**0.003**		**0.031**		
	Hygrophilous mesofauna	13.15%	0.765	0.711	0.831								

Note: pH = soil pH value; SBD = soil bulk density; AN = ammonium N; AP = available P; AK = available K; WC = soil water content; AT = air temperature; ST = soil temperature (*p* < 0.05). CCA was used for all soil animal groups in autumn, as well as macrofauna and microarthropods in summer; RDA was used for hygrophilous mesofauna in summer (the longest gradient was <3).

## Appendix H

**Table A7:** *p* values of indicator taxa for revetment types and distances in two seasons.

Family	Entire Soil Fauna Community												Macrofauna												Microarthropods												Hygrophilous Mesofauna											
	Summer						Autumn						Summer						Autumn						Summer						Autumn						Summer						Autumn					
	Type			Distance			Type			Distance			Type			Distance			Type			Distance			Type			Distance			Type			Distance			Type			Distance			Type			Distance		
	NR	MR	HR	0	5	10	NR	MR	HR	0	5	10	NR	MR	HR	0	5	10	NR	MR	HR	0	5	10	NR	MR	HR	0	5	10	NR	MR	HR	0	5	10	NR	MR	HR	0	5	10	NR	MR	HR	0	5	10
Anthocoridae													0.028																																			
Theridiidae	0.031												0.020																																			
Subulinidae		0.002												0.004																																		
Geophilidae		0.023												0.021																																		
Julidae		0.011												0.006																																		
Leptanillinae		0.011												0.008																																		
Spirobolidae		0.033												0.031																																		
Campodeidae		0.022												0.033																																		
Onychiuridae	0.001																								0.001																							
Isotomidae	0.013																								0.012																							
Hypogastruridae		0.001																								0.001																						
Oribatulidae		0.020																								0.022																						
Pratylenchidae	0.003			0.001			0.032																														0.003			0.001			0.023			0.023		
Dorylaimidae	0.001																																				0.001											
Rhabditidea	0.029			0.022																																	0.023			0.035								
Paratylenchidae	0.031			0.007																																	0.029			0.007								
Heteroderidae	0.002																																				0.004											
Aphelenchoidea			0.001	0.015																																			0.001	0.010								
Ctenidae							0.012												0.004																													
Staphylinidae							0.031												0.025																													
Kalotermitidae								0.005												0.005																												
Tetrigoidea								0.004												0.013																												
Cydnidae								0.031												0.031																												
Zetorchestidae								0.009																								0.006																
Malaconothridae									0.013																								0.012															
Tylenchidae				0.002						0.008																														0.004								
Plectidae				0.024																																				0.024								
Japygidae											0.006												0.009																									
Grylloidea											0.033												0.036																									
Total number of indicator families	8	8	1	6			3	4	1	1	2		2	6					2	3			2		2	2						1	1				5		1	6			1			1		

## Appendix I

**Table A8:** Numbers of indicator taxa for revetment types and distances in two seasons.

Season	Entire Soil Fauna Community						Macrofauna						Microarthropods						Hygrophilous Mesofauna					
	Type			Distance			Type			Distance			Type			Distance			Type			Distance		
	NR	MR	HR	0	5	10	NR	MR	HR	0	5	10	NR	MR	HR	0	5	10	NR	MR	HR	0	5	10
Summer	8	8	1	6	0	0	2	6	0	0	0	0	2	2	0	0	0	0	5	0	1	6	0	0
Autumn	3	4	1	1	2	0	2	3	0	0	2	0	0	1	1	0	0	0	1	0	0	1	0	0
